# Supplementary material for: Associations between Pleurisy and the Main Bacterial Pathogens of the Porcine Respiratory Diseases Complex (PRDC)
Source: Animals (Basel). 2023 Apr 27;13(9):1493. doi: 10.3390/ani13091493 (PMC10177087; doi:10.3390/ani13091493)
Supplement: Supplementary file 1 [file animals-13-01493-s001.zip › animals-2301737-supplementary.pdf]

Table S1: Parameters of multiplex qPCR assays based on *omIA* gene for *A. pleuropneumoniae*. *p102* for *M. hyopneumoniae* and *kmt1* for *P. multocida*

Pathogen/Target	Plate	Fluorophore	E (%)	R^2	Slope	y-intercept
	1		102.8	0.992	3.212	35.973
A. pleuropneumoniae/omIA	2	Cy5	99.2	0.991	3.399	36.313
	3		101	0.994	3.298	35.37
	1		102.1	0.993	3.272	37.9
M. hyopneumoniae/ p102	2	FAM	93.5	0.991	3.369	37.156
	3		95.7	0.999	3.489	36.62
	1		95.9	0.993	3.423	36.255
P. multocida/kmt1	2	TxRed	97.1	0.998	3.568	37.145
	3		96.8	0.997	3.401	36.665

Table S2: Parameters of qPCR assays based on *gdh* for *S. suis* and *infB* gene for *G. parasuis*.

Pathogen/Target	Plate	Efficiency (%)	R^2	Slope	y-intercept
	1	97.3	0.996	3.388	35.675
S. suis/gdh	2	97.2	0.996	3.383	36.106
	3	95.2	0.992	3.44	35.481
	1	100.3	0.996	3.315	34.44
G. parasuis/infB	2	98.6	0.994	3.355	36.524
	3	95.50%	0.989	3.436	34.321
